# Gut Microbiota Regulates the Sympathetic Nerve Activity and Peripheral Serotonin Through Hypothalamic MicroRNA-204 in Order to Increase the Browning of White Adipose Tissue in Obesity

**DOI:** 10.7759/cureus.21913

**Published:** 2022-02-04

**Authors:** Adam Kassan, Karima Ait-Aissa, Modar Kassan

**Affiliations:** 1 Pharmacy, West Coast University, Los Angeles, USA; 2 Internal Medicine, University of Iowa, Iowa City, USA; 3 Physiology, University of Tennessee Health Science Center, Memphis, USA

**Keywords:** enterochromaffin cells, 5-ht, browning of white adipose tissue, hypothalamic mir-204, obesity

## Abstract

The prevalence of obesity is increasing worldwide, and novel therapeutic strategies such as enhancement of thermogenic pathways in white adipose tissue (WAT) are gaining more attention. The gut/brain axis plays an essential role in promoting the browning of WAT. However, the mechanism by which this axis regulates WAT function is not fully understood. On the other hand, the role of microRNAs (miRNAs) in the control of WAT browning has already been established. Therefore, understanding the communication pathways linking the gut/brain axis and miRNAs might establish a promising intervention for obesity.

Our published data showed that microRNA-204 (miR-204), a microRNA that plays an important role in the control of the central nervous system (CNS) and the pathogenesis of obesity, is affected by gut dysbiosis. Therefore, miR-204 could be a key element that controls the browning of WAT by acting as a potential link between the gut microbiota and the brain. In this review, we summarized the current knowledge about communication pathways between the brain, gut, and miR-204 and examined the literature to discuss potential research directions that might lead to a better understanding of the mechanisms underlying the browning of WAT in obesity.

## Introduction and background

The prevalence of obesity and associated metabolic disorders is increasing worldwide, and novel therapeutic strategies are needed. Recently, increasing energy expenditure through thermogenesis of white adipose tissue (WAT), known as browning/beiging of WAT, has been recognized as a therapeutic strategy for weight loss in humans [1]. Over the last decade, extensive studies in the area of metabolic disorders pointed toward gut microbiota as an important player in regulating the browning of WAT [2]. Another component that plays an important role in regulating WAT thermogenesis is the brain [3]. The gut/brain axis is a bidirectional communication between the central nervous system (CNS) and the gastrointestinal tract [4]. Several peripheral signals relaying information about energy status act in the brain, particularly the hypothalamus, to regulate the browning of WAT [5]. It has been shown that these signals, responsible for the control of systemic energy balance, are regulated by hypothalamic miRNAs, key regulators of gene expression [6,7]. Additionally, gut microbiota can affect the expression of central miRNAs [8,9]. Thus, any miscommunication between the gut and the brain could affect these peripheral signals and might lead to metabolic disorders.

Interestingly, our published data showed that microRNA-204 (miR-204), a microRNA that plays an important role in the CNS function [10] and the pathophysiology of obesity [11], is indeed affected by gut dysbiosis [11]. Therefore, we speculated that miR-204 could be a key element connecting the gut microbiota to the brain in order to control the WAT thermogenesis during obesity. In this review, we will summarize what has been published about the communication pathways between the brain, gut, and miR-204. Based on the current knowledge, we will also examine potential research directions that might lead to a better understanding of the mechanisms underlying the browning of WAT in obesity. Based on the different findings in the literature, we will suggest a potential hypothesis by which hypothalamic miR-204 could emerge as a key player in regulating obesity through the browning of WAT.

## Review

Hypothalamic miR-204 and sympathetic nerve activity

Hypothalamus is one of the most important areas in the CNS that regulate energy homeostasis [12], but the exact mechanism by which the hypothalamus regulates energy expenditure is not well established. Lately, it has been shown that the sympathetic nervous system (SNS) plays a key role in promoting the browning of WAT [13]. Recent scientific publications have reported that many key elements in the hypothalamus could affect the SNS [14,15]. One important element is the nicotinamide adenine dinucleotide (NAD+)‐dependent histone/protein deacetylase or simply sirtuin 1 (SIRT1), which is widely expressed in many tissues including the hypothalamus [16]. A study by Liu et al. showed that inhibition of hypothalamic SIRT1 led to a decrease in the sympathetic nerve activity (SNA) [14]. Brain-derived neurotrophic factor (BDNF), another element that is expressed in the hypothalamus, has also been shown to regulate SNA [15,17]. Recent studies showed that increased levels of BDNF in the paraventricular nucleus of the hypothalamus (PVN) led to increased sympathetic activity [15,17]. 

Furthermore, we and others have already demonstrated that miR-204 can target and downregulate SIRT1 [11,18], while other studies showed that miR-204 can target and downregulate BDNF [19-23]. We and others showed that miR-204 increases during obesity [11,24-26], whereas SIRT1 and BDNF are reduced in obesity [27-30]. Additionally, it has already been shown that miR-204 is expressed in the hypothalamus [31]. Based on this literature evidence, we can speculate that increased hypothalamic miR-204 targets and downregulates SIRT1 and BDNF, which in turn could lead to a decrease in the SNA.

SNA, enterochromaffin cells (ECs), peripheral serotonin (5-HT), and thermogenesis

Peripheral serotonin (5-HT) (80%-90% of the total body content of serotonin) is synthesized in the gut predominately by the enterochromaffin cells (ECs) from tryptophan-by-tryptophan hydroxylase 1 (Tph1) [32-35]. Interestingly, the release of 5-HT from the ECs, the most abundant enteroendocrine cell subtype of the colon [36], is under the control of the autonomous nervous system [37]. ECs are known to respond well to several physiological compounds such as norepinephrine (NE) [38], which is released by sympathetic nerve fibers in the gut [39], most specifically, the colon [40]. ECs are known to express the alpha 2 (α2) adrenergic receptor [38,41]. Once NE binds to its α2 receptor, it leads to a reduction in cyclic adenosine monophosphate (cAMP) [42]. It is important to mention that cAMP is critical for Tph1 function [43,44] and stability [45]; so, a decrease in cAMP will affect the Tph1 function [43,44], leading to less peripheral 5-HT release [45]. Additionally, studies performed in vivo indicate that α adrenergic agonists inhibit the release of peripheral 5-HT from ECs [41]. It is well known that during obesity, peripheral 5-HT is increased [46] and blunts the thermogenesis by negatively regulating the sensitivity of adipose tissues to β-adrenergic stimulation [47] that is known to stimulate peroxisome proliferator-activated receptors gamma (PPARg) and peroxisome proliferator-activated receptor-gamma coactivator 1α (PGC1α), which are master regulators of uncoupling protein 1 (UCP1)-mediated thermogenesis [48,49]. Interestingly, downregulation of peripheral 5-HT reduced obesity and promoted the browning of adipose tissue [47,50-51].

We already explained in the previous section, and based on literature findings, that an increase in miR-204 in obesity could lead to downregulation of SIRT1 and BDNF and, therefore, to a decrease in the SNA. Taken all together, we can hypothesize that increased hypothalamic miR-204 in obesity will lead to less NE binding to α2 receptors in ECs. This eventually will increase cAMP that can activate and stabilize Tph1 leading to more peripheral 5-HT release and, therefore, will destabilize the thermogenesis pathway in WAT. Thus, the hypothalamic miR-204/ peripheral 5-HT axis might be effective for treating obesity and increasing thermogenesis.

Microbiota, miR-204, and peripheral serotonin

**Figure 1:**
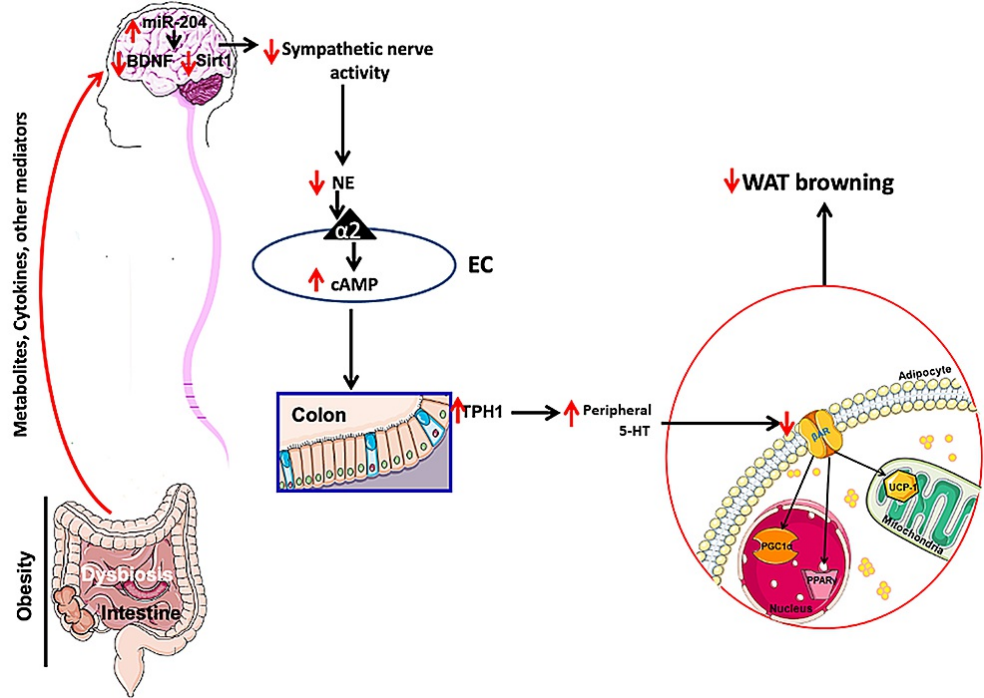
Scheme summarizing the hypothesis in this review miR-204, MicroRNA-204; NAD+, nicotinamide adenine dinucleotide; SIRT1, sirtuin 1; BDNF, brain-derived neurotrophic factor; NE, norepinephrine; α2, alpha 2; Tph1, tryptophan hydroxylase 1; 5-HT, serotonin; PPARg, peroxisome proliferator-activated receptors gamma; PGC1α, peroxisome proliferator-activated receptor-gamma coactivator 1α; UCP1, uncoupling protein 1; WAT, white adipose tissue.

Manipulating bacterial composition using antibiotics or germ-free mice (GFM) showed that depletion of bacteria significantly increased the browning of WAT [52,53]. Additionally, mice on antibiotics or GFM showed a decreased level of peripheral 5-HT [54,55], and GFM modulated the sympathetic activity [56]. For instance, it has been shown that antibiotics increased the BDNF [57]. Moreover, GFM or treatment with antibiotics [11] showed decreased levels of miR-204 and increased levels of SIRT1 [11]. Interestingly, recolonization of these mice or fecal transplant from obese mice reversed all these parameters (Figure 1) [11].

## Conclusions

Taken all together and based on the literature discussed above, we can hypothesize that obesity could increase hypothalamic miR-204 leading to decreased levels of SIRT1 and BDNF, which in turn will lead to a decreased SNA. A decrease in SNA means that less NE is binding to α2 receptors in ECs, leading to more cAMP that stabilizes and activates Tph1 to produce more peripheral 5-HTs that blunt the thermogenesis by negatively regulating the sensitivity of adipose tissues to β-adrenergic stimulation. Decreasing the β-adrenergic stimulation will lead to less PPARg and PGC1α that are master regulators of UCP1-mediated thermogenesis, thus producing less thermogenesis.
